# Laparoscopic versus open distal gastrectomy for elderly patients with advanced gastric cancer: a retrospective comparative study

**DOI:** 10.1186/s12957-022-02819-4

**Published:** 2022-11-09

**Authors:** Hung-Hsuan Yen, Chi-Chuan Yeh, I-Rue Lai

**Affiliations:** 1 Department of Surgery, National Taiwan University Hospital Hsin-Chu Branch, Hsinchu County, Taiwan; 2 Department of Surgery, National Taiwan University Hospital, Taipei, Taiwan; 3 Graduate Institute of Anatomy and Cell Biology, College of Medicine, National Taiwan University, Taipei, Taiwan

**Keywords:** Advanced gastric cancer, Elderly, Gastrectomy, Laparoscope, Complication

## Abstract

**Background:**

Laparoscopic radical distal gastrectomy (LDG) has been more frequently performed for locally advanced distal gastric cancer (AGC) than open distal gastrectomy (ODG). However, the benefits of LDG for elderly AGC patients (AGC-lap) remain unclear.

**Methods:**

Patients aged ≥ 70 years who underwent D2 distal gastrectomy from July 2014 to July 2021 were enrolled consecutively. Perioperative parameters, pathological features, and oncological outcomes of AGC-lap patients (*n* = 39) were compared with those of elderly AGC patients receiving ODG (AGC-open; *n* = 37) and elderly early gastric cancer patients receiving LDG (EGC-lap; *n* = 41) respectively.

**Results:**

The median age of all AGC patients was 77 years, and 28% of them had an Eastern Cooperative Oncology Group score ≥ 2. Most of the perioperative and pathological features (including the number of lymph nodes harvested) were similar between the AGC-lap and AGC-open groups. AGC-lap patients had longer median operative times (215 min versus 192 min) but significantly less surgical complications (10.3% versus 37.8%) and shorter median hospital stays (11 days versus 13 days) than did AGC-open patients (all *p* < 0.05). The 3-year recurrence-free and overall survival was 66.2% and 88.8% in the AGC-lap group and 51% and 66.3% in the AGC-open group (both *p* = 0.1). The perioperative features, including operative time, number of lymph nodes harvested, hospital stay, and complication rates, were similar between the AGC- and EGC-lap groups.

**Conclusions:**

LDG was safely and effectively performed in elderly AGC patients, resulting in faster recovery and a lower complication rate than ODG, without compromising oncological outcomes.

## Introduction

Gastric cancer (GC) is the sixth most common cancer and fourth most common cause of cancer-related death worldwide [1]. Although less common in some Western countries, surgical resection with a D2 gastrectomy is a standard initial treatment for early GC (EGC) and locally advanced GC (AGC) in Eastern countries [2, 3]. With improvements in surgical techniques and devices and perioperative care, laparoscopic distal gastrectomy (LDG) has evolved from a laparoscopy-assisted to a fully laparoscopic method and is now a well-established alternative to open distal gastrectomy (ODG) for EGC in the lower stomach, providing advantages including reduced wound pain, improved pulmonary function, and earlier gastrointestinal tract function recovery [4–6].

The long-term oncological benefits of LDG (compared to ODG) for EGC have been determined by many studies, including two key randomized controlled trials (RCTs), JCOG0912 and KLASS-01 [4, 7–11]. However, the advantages of LDG for AGC are still inconclusive and are not yet generally accepted. Although the recently published meta-analysis and two RCTs, CLASS-01 and KLASS-02 trials, strengthen the argument for using LDG for AGC, patients ≥ 70 years old were often a minority and those ≥ 75 years old were ineligible to enroll in these studies [12–15]. Previous RCTs were unable to show enough evidence on elderly AGC patients receiving LDG, and existing literature is limited to a few retrospective and ongoing prospective studies and is therefore insufficient [16–19].

As the world is aging, cancer burden is growing among older adults [20]. According to the most recent nationwide survey, approximately 30% of GC cases in Korea were in patients ≥ 70 years old [21]. In addition, age ≥ 70 years is also a critical risk factor for postoperative complications and longer hospital stays [22, 23]. Decisions about surgery in elderly patients are difficult because aging is often associated with shorter life expectancy, less functional reserves, and more comorbidities [24, 25]. The advantages of laparoscopic surgery in elderly patients are considered to outweigh the disadvantages generally; however, whether these benefits remain in LDG for AGC, a type of surgery that requires meticulous lymph node dissection and possibly longer pneumoperitoneum time, is still unclear [26–29].

Thus, the purpose of this study was first to evaluate the surgical and oncological outcomes of elderly patients (≥ 70 years old) with AGC in the distal stomach receiving LDG or ODG, and secondly, to compare short-term surgical outcomes for elderly AGC and EGC patients receiving LDG. Finally, we aimed to make up for the deficiency of this important but often neglected issue via sharing our real-world data.

## Methods

### Identification of the study cohort and study design

**Fig. 1:**
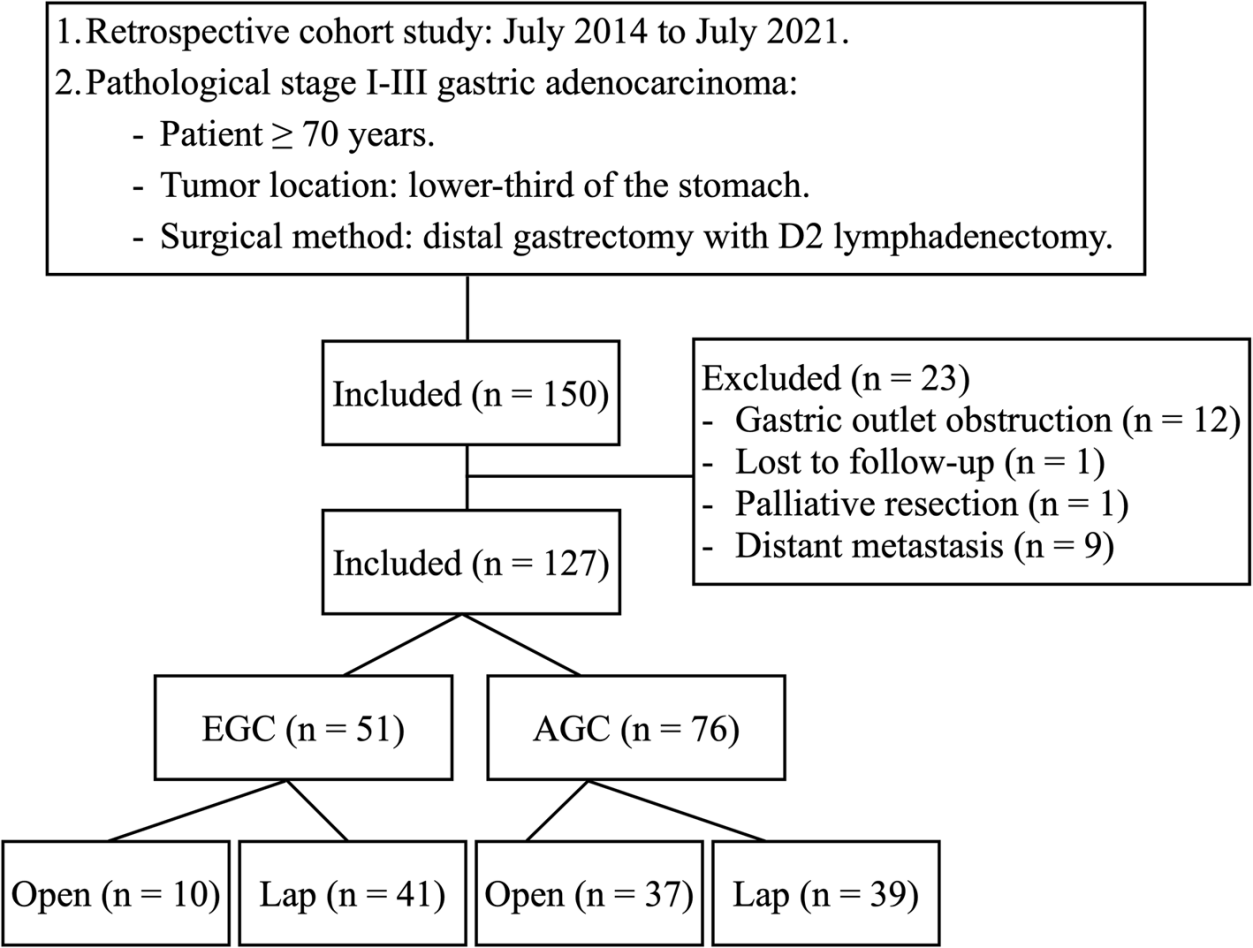
Flow chart of the inclusion criteria for the study. Abbreviations: EGC, early gastric cancer; AGC, advanced gastric cancer; Lap, laparoscopic surgery

We consecutively recruited GC patients aged ≥ 70 years with tumors in the lower third of the stomach and receiving standard D2 distal gastrectomy. All the D2 distal gastrectomies were performed by the same surgical team, led by an experienced surgeon (I.-R. L.), at a tertiary referral medical center from July 2014 to July 2021. Patients who presented with total gastric outlet obstruction and/or tumor invasion to adjacent organs (clinical T4b lesion), were lost to follow-up (follow-up time < 1 month), received palliative resection, or had distant metastatic disease were excluded from the study (Fig. 1). The study was approved by the Institutional Review Board of National Taiwan University Hospital (NTUH-REC No.: 202101037RINB).

**Fig. 2:**
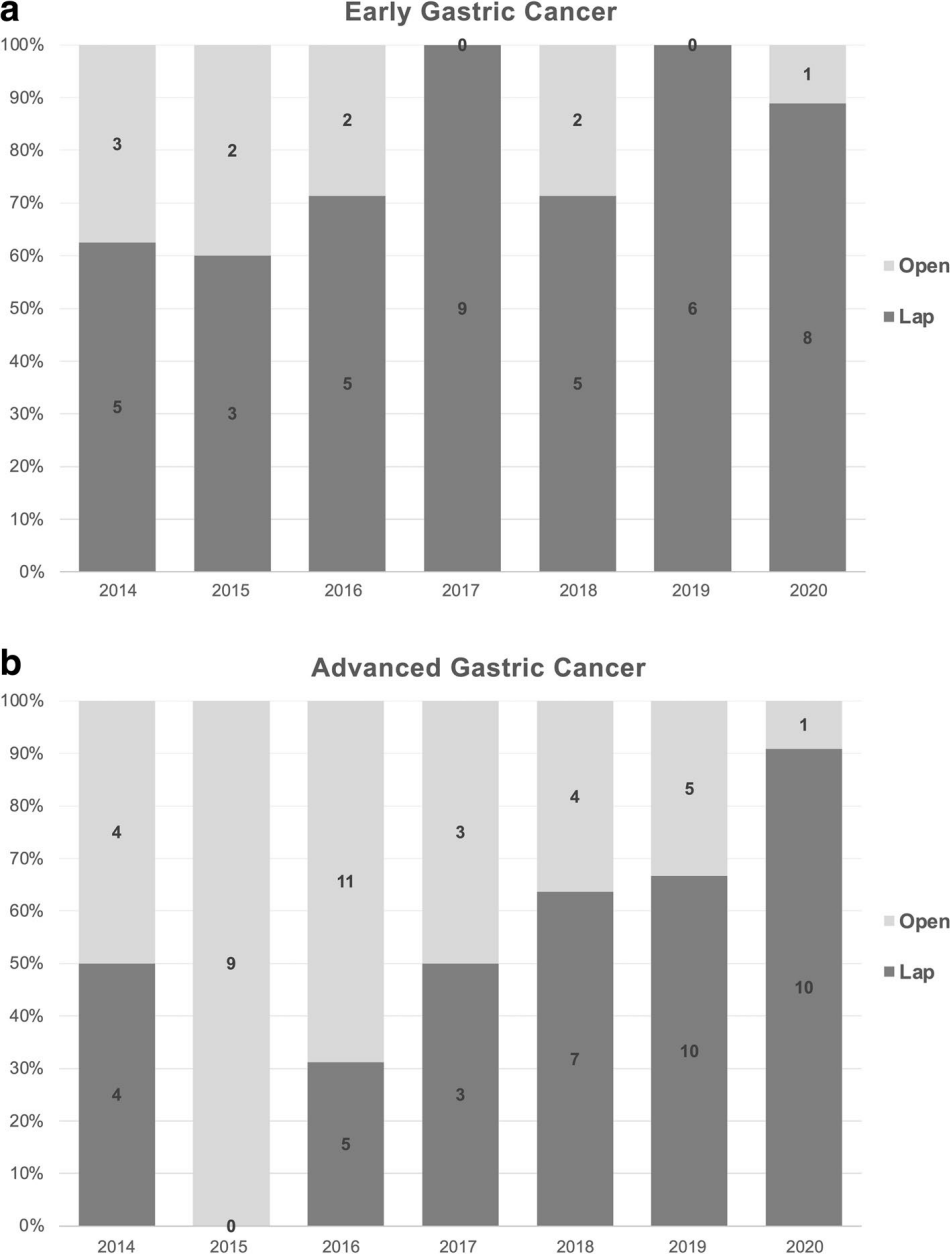
Annual distribution of laparoscopic and open distal gastrectomies for early (**a**) and advanced (**b**) gastric cancers from 2014 to 2020. Abbreviation: Lap, laparoscopic surgery

Surgical margins and the extent of lymph node dissection strictly followed the guidelines released by the Japanese Gastric Cancer Association [2]. In brief, gross resection margins were > 2 cm for clinical T1 tumors, > 3 cm for ≥ T2 Borrmann type 1 and 2 tumors, and > 5 cm for ≥ T2 Borrmann type 3 and 4 tumors, proximally. The lymph node stations 1, 3, 4sb, 4d, 5, 6, 7, 8a, 9, 11p, 12a, and 12p were dissected. Typically, only total/nearly total gastric outlet obstruction and/or adjacent organ invasion (clinical T4b lesion) were considered absolute contraindications to LDG (Fig. 2).

The patients were classified into EGC (pathological stage I) and AGC (pathological stage II or III) groups, according to the eighth edition of the American Joint Committee on Cancer staging manual [30], and these two groups were further divided into laparoscopic (lap) and open groups based on the surgical method used. We focus here on comparisons between the AGC-lap and AGC-open groups and between the AGC- and EGC-lap groups.

### Perioperative assessment, postoperative care, and outpatient follow-up

All patients received a comprehensive preoperative evaluation, including a physical examination, blood test (complete blood count, chemistry panels, clot-based activity assays, and tumor markers), chest plain film, abdominal computed tomography scan, and esophagogastroduodenoscopy (EGD), prior to surgery. Tumor location (lower third of the stomach) and pathology (adenocarcinoma) were always confirmed during preoperative EGD. Cardiac sonography and lung function tests were only performed for patients with a medical history or physical findings significant for heart and/or pulmonary diseases. The patients’ performance and physical status were evaluated at admission using the Eastern Cooperative Oncology Group (ECOG) performance-status scale and the American Society of Anesthesiologists (ASA) physical-status classification system, respectively. The Charlson comorbidity index (CCI) was used to assess preoperative comorbid conditions [31].

ODG was conducted via a midline laparotomy from the subxiphoid to the umbilicus. LDG was conducted via four-port laparoscopy, as described previously [4]. Billroth I anastomosis was the preferred reconstruction method, followed by Billroth II and Roux-en-Y reconstruction if the tension of gastroduodenostomy persisted after Kocher’s maneuver. A 7-mm Jackson–Pratt drain was routinely placed in the right subhepatic and left subphrenic space, irrespective of surgical method. Patients were transferred to the intensive care unit for early postoperative care, according to the anesthesiologist’s judgment.

Nasogastric tubes were routinely used for decompression until postoperative day 5, and oral intake was initiated with clear liquids, followed by rice soup, rice porridge, and finally regular post-gastrectomy diet on postoperative days 6–9 if recovery remained uneventful. If patients were nauseous or complained of abdominal fullness, the diet protocol was paused and prokinetic agents were given. Nasogastric tubes were sometimes reinserted for decompression. The diet protocol was resumed once the abdominal symptoms had resolved and flatulence was noted. The Jackson–Pratt drains were removed simultaneously or sequentially on postoperative days 9–11 if there were no signs of leakage. Patients with no adverse events were usually discharged on postoperative days 10–12.

The outpatient follow-up protocol was followed as previously described and complied with guidelines released by the Japanese Gastric Cancer Association [2, 3]. Computed tomography scan, EGD, or outpatient visit scheduling was moved forward or increased in frequency if patients exhibited signs of recurrence. Long-term follow-up data were acquired from electronic medical records and databases maintained by the Cancer Administration and Coordination Center in our hospital.

### Statistical analysis

The cut-off value of the ECOG score and ASA classification to categorize patients was set at > 1 and > 2, respectively, because those with ECOG score > 1 and/or ASA class > 2 were considered at higher risk and often excluded from RCTs [12, 14]. The geriatric nutritional risk index (GNRI) [32] was calculated, and those with GNRI ≤ 98 were considered to have an increased risk of morbidity and mortality during hospitalization. The prognostic nutrition index (PNI) was calculated, and those with PNI ≤ 48 were considered to have a lower 5-year overall survival probability [33, 34]. Postoperative complications were graded based on the Clavien–Dindo classification, and grade 3 or higher complications were considered to be clinically significant [35]. Recurrence-free survival (RFS) was defined as the period between the surgery and the first event (all-cause death, recurrence of gastric cancer, or occurrence of a second cancer). Overall survival (OS) was defined as the period between the surgery and all-cause death. Patients lost to follow-up were censored.

Continuous data are expressed as medians (interquartile range [IQR]). Categorical variables are expressed as numbers (percentages). The Wilcoxon rank–sum test (Mann–Whitney *U* test) was used to analyze quantitative variables, and chi-squared or Fisher’s exact tests were used for qualitative variables, as deemed appropriate. We assessed the time-to-event endpoint using the Kaplan–Meier method and estimated the hazard ratio (HR) and its two-sided 95% confidence interval (CI) using the Cox regression model. The two survival distributions were compared using the log-rank test. Univariable analysis compared, first, the AGC-lap and AGC-open groups and, second, the AGC- and EGC-lap groups, respectively. Survival analysis compared the AGC-lap and AGC-open groups. All statistical tests were two-sided, and a *p*-value < 0.05 denoted statistical significance. Statistical analysis was performed using R version 3.4.0 (R Core Team, 2017).

## Results

### Preoperative basic characteristics and treatments

We identified 150 GC patients ≥ 70 years old who received distal gastrectomy by the same surgical team between July 2014 and July 2021 and tracked them in our prospectively maintained database. Patients with gastric outlet obstruction (*n* = 12), who were lost to follow-up (*n* = 1), were receiving palliative resection (< D2 dissection; *n* = 1), or presented with metastatic disease (*n* = 9) were excluded, leaving 127 patients who were included. There were 39 patients in the AGC-lap group, 37 in the AGC-open group, and 41 in the EGC-lap group (Fig. 1). After 2016, a greater proportion of AGC cases received LDG, as did almost all EGC cases (Fig. 2).

The median age at the time of surgery was 77 years (IQR: 73–83.5 years), 76 years (IQR: 73–81 years), and 77 years (IQR: 74–80 years) in the AGC-lap, AGC-open, and EGC-lap groups, respectively. Baseline characteristics (body mass index, CCI, and percentage with ECOG score > 1, ASA classification > 2, GNRI ≤ 98, and PNI ≤ 48) were similar between the AGC-lap and AGC-open groups and between the AGC- and EGC-lap groups (Tables 1 and 3). There was a somewhat higher prevalence of clinical node-positive GC in the AGC-open (70%) than in the AGC-lap group (51%; *p* = 0.11).

**Table 1:** Demographics and treatments of elderly AGC patients who underwent distal gastrectomy

	Advanced gastric cancer						
	All	(*n* = 76)	Open	(*n* = 37)	Lap	(*n* = 39)	*p*
***Demographics and preoperative features***							
Sex (male)	45	(59)	18	(49)	27	(69)	0.10
Age (years)	77	(73–83)	76	(73–81)	77	(73–83.5)	0.40
BMI (kg/m^2^)	24.2	(21.9–26.8)	25	(22.3–27.4)	23.9	(21.9–26.1)	0.40
Charlson comorbidity index	1	(1–2)	1	(1–2)	1	(1–2)	0.40
ECOG score (> 1)	21	(28)	10	(27)	11	(28)	0.99
ASA classification (> 2)	17	(22)	8	(22)	9	(23)	0.99
GNRI (≤ 98)	17	(23)	7	(19)	10	(26)	0.60
PNI (≤ 48)	36	(48)	21	(57)	15	(39)	0.20
Clinical N+ stage	46	(61)	26	(70)	20	(51)	0.11
***Surgical procedure and adjuvant therapy***							
Anastomotic type							0.011*
Billroth I	42	(55)	15	(41)	27	(69)	
Billroth II	30	(39)	18	(49)	12	(31)	
Roux-en-Y	4	(5.3)	4	(11)	0	(0)	
Combined resection	8	(11)	3	(8.1)	5	(13)	0.7
Adjuvant chemotherapy	31	(41)	13	(35)	18	(46)	0.40

Data are presented as median (interquartile range) or *n* (%)

*AGC* advanced gastric cancer, *Lap* laparoscopic surgery, *BMI* body mass index, *ECOG* Eastern Cooperative Oncology Group, *ASA* The American Society of Anesthesiologists, *GNRI* geriatric nutritional risk index, *PNI* prognostic nutrition index

*p < 0.05, Lap versus Open

D2 lymphadenectomy was conducted for all the patients included in this study. Billroth I reconstruction was performed most frequently in the EGC-lap group (85%), followed by the AGC-lap group and the AGC-open group (69% versus 41%; *p* = 0.011) (Tables 1 and 3). Adjuvant chemotherapy was conducted in 35% of AGC-open patients and 46% AGC-lap patients (*p* = 0.40) (Table 1).

### Pathological features

All pathological features were similar between AGC-lap and AGC-open patients, except that perineural invasion was more frequent in the AGC-open (68%) than in the AGC-lap group (36%; *p* = 0.007) (Table 2). Median tumor size was slightly larger in the AGC-open (5.5 cm; IQR: 4–7 cm) than in the AGC-lap group (4.5 cm; IQR: 3.15–6 cm; *p* = 0.10), and the median number of harvested lymph nodes was also slightly larger in the AGC-open group (42; IQR: 29–52) than in the AGC-lap group (37; IQR: 30–45; *p* = 0.30). The percentage of pathological T3 and T4 lesions was similar; however, the proportion of pathological N3 lesions was slightly higher in the AGC-open group (41%) than in the AGC-lap group (28%; *p* = 0.20).

**Table 2:** Pathological features and outcomes of elderly AGC patients who underwent distal gastrectomy

	Advanced gastric cancer						
	All	(*n* = 76)	Open	(*n* = 37)	Lap	(*n* = 39)	*p*
***Pathological features***							
Site							0.10
Pylorus or antrum	70	(92)	32	(86)	38	(97)	
Low body	6	(7.9)	5	(14)	1	(2.6)	
Size (cm)	5.0	(3.22–6.5)	5.5	(4–7)	4.5	(3.15–6)	0.10
Bormann type							0.075
Early gastric cancer	1	(1.3)	0	(0)	1	(2.6)	
I or II	17	(22)	5	(14)	12	(31)	
III or IV	58	(76)	32	(86)	26	(67)	
Lauren classification							0.99
Intestinal type	56	(74)	28	(76)	28	(72)	
Diffuse type	12	(16)	5	(14)	7	(18)	
Mixed type	8	(11)	4	(11)	4	(10)	
Cell differentiation							0.70
Well differentiated	8	(11)	4	(11)	4	(10)	
Moderately differentiated	39	(51)	17	(46)	22	(56)	
Poorly or undifferentiated	29	(38)	16	(43)	13	(33)	
LN metastasis (number)	4	(1–8)	5	(1–10)	4	(2–8)	0.90
LN harvested (number)	39	(29–48)	42	(29–52)	37	(30–45)	0.30
Lymphovascular invasion	53	(70)	26	(70)	27	(69)	0.99
Perineural invasion	39	(51)	25	(68)	14	(36)	0.007*
Pathological T stage (AJCC 8th)							0.5
1	5	(6.6)	1	(2.7)	4	(10)	
2	15	(20)	6	(16)	9	(23)	
3	34	(45)	18	(49)	16	(41)	
4	22	(29)	12	(32)	10	(26)	
Pathological N stage (AJCC 8th)							0.20
0	10	(13)	7	(19)	3	(7.7)	
1	17	(22)	7	(19)	10	(26)	
2	23	(30)	8	(22)	15	(38)	
3	26	(34)	15	(41)	11	(28)	
Pathological stage (AJCC 8th)							0.8
II	36	(47)	17	(46)	19	(49)	
III	40	(53)	20	(54)	20	(51)	
***Short-term and long-term outcomes***							
Operative time (min)	206	(178–228)	192	(165–212)	215	(190–238)	0.006*
Blood loss (mL)	100	(0–200)	100	(0–200)	100	(0–150)	0.059
Complications (≥ grade 3)	10	(13)	5	(14)	5	(13)	0.99
Length of postoperative hospitalization (d)	12	(10–16)	13	(11–22)	11	(10–14.5)	0.042*
Follow-up (mo)	22	(12–38)	27	(15–44)	15	(10–32)	0.03*
Three-year RFS (%)	58.6	(47.9–71.7)	51.0	(37.1–70.1)	66.2	(51.1–85.7)	0.10

Data are presented as median (interquartile range) or *n* (%). Three-year RFS is presented as % (95% confidence interval)

*AGC* advanced gastric cancer, *Lap* laparoscopic surgery, *LN* lymph node, *AJCC* American Joint Cancer Committee, *RFS* recurrence-free survival

*p < 0.05, Lap versus Open

On the other hand, pathological features, including tumor size, percentage of Borrmann types, number of metastatic lymph nodes, proportion of lymphovascular invasion, perineural invasion, and pathological stages, differed significantly between the AGC-lap and EGC-lap groups as expected, while the number of lymph nodes harvested was similar (Table 3).

**Table 3:** Demographics and perioperative features of elderly EGC and AGC patients who underwent laparoscopic distal gastrectomy

	Laparoscopic surgery				
	EGC	(*n* = 41)	AGC	(*n* = 39)	*p*
***Demographics, anthropometrics, and preoperative features***					
Sex (male)	22	(54)	27	(69)	0.20
Age (years)	77	(74–80)	77	(73–83.5)	0.60
BMI (kg/m^2^)	24.2	(22.8–26.3)	23.9	(21.9–26.1)	0.50
Charlson comorbidity index	2	(1–3)	1	(1–2)	0.20
ECOG score (> 1)	7	(17)	11	(28)	0.30
ASA classification (> 2)	7	(17)	9	(23)	0.60
GNRI (≤ 98)	7	(17)	10	(26)	0.40
PNI (≤ 48)	14	(34)	15	(39)	0.60
Clinical N+ stage	5	(12)	20	(51)	<0.001*
***Pathological features***					
Site					0.99
Pylorus or antrum	40	(98)	38	(97)	
Low body	1	(2.4)	1	(2.6)	
Size (cm)	2.6	(2–3)	4.5	(3.15–6)	<0.001*
Bormann type					<0.001*
Early gastric cancer	21	(51)	1	(2.6)	
I or II	10	(24)	12	(31)	
III or IV	10	(24)	26	(67)	
Lauren classification					0.99
Intestinal type	28	(68)	28	(72)	
Diffuse type	8	(20)	7	(18)	
Mixed type	4	(9.8)	4	(10)	
LN metastasis (number)	0	(0–0)	4	(2–8)	<0.001*
LN harvested (number)	31	(24–40)	37	(30–45)	0.084
Pathological stage (AJCC 8th)					<0.001*
I	41	(100)	0	(0)	
II	0	(0)	19	(49)	
III	0	(0)	20	(51)	
***Surgical and postsurgical features***					
Anastomotic type					0.11
Billroth I	35	(85)	27	(69)	
Billroth II	6	(15)	12	(31)	
Combined resection	5	(12)	5	(13)	0.99
Operative time (min)	203	(186–219)	215	(190–238)	0.10
Blood loss (mL)	100	(0–100)	100	(0–150)	0.80
Complications (≥ grade 3)	2	(4.9)	5	(13)	0.30
Length of postoperative hospitalization (d)	11	(10–13)	11	(10–14.5)	0.50
Follow-up (mo)	35	(13–59)	15	(10–32)	0.007*

Data are presented as median (interquartile range) or *n* (%)

*EGC* early gastric cancer, *AGC* advanced gastric cancer, *BMI* body mass index, *ECOG* Eastern Cooperative Oncology Group, *ASA* The American Society of Anesthesiologists, *GNRI* geriatric nutritional risk index, *PNI* prognostic nutrition index, *LN* lymph node, *AJCC* American Joint Cancer Committee

*p < 0.05, EGC-lap versus AGC-lap

### Short-term and long-term outcomes

Operative time was significantly longer in the AGC-lap group (215 min; IQR: 190–238 min) than in the AGC-open group (192 min; IQR: 165–212 min; *p* = 0.006; Table 2). Rates of combined resection, grade 3 or higher complications, and amount of blood loss were similar. The length of postoperative hospitalization was significantly shorter in the AGC-lap group (11 days; IQR: 10–14.5 days) than in the AGC-open group (13 days; IQR: 11–22 days; *p* = 0.042). In contrast, the EGC-lap group was similar to the AGC-lap group in terms of anastomotic type, operative time, and length of postoperative hospitalization (Table 3).

All-grade total and surgical complications occurred more frequently in the AGC-open group (43.2% and 37.8%) than in the AGC-lap group (17.9% and 10.3%; both *p* < 0.05). AGC-open patients tended to have more ileus/gastroparesis (10.8% versus 0%; *p* < 0.05) and intra-abdominal abscesses (13.5% versus 0%; *p* < 0.05) than AGC-lap patients. Nevertheless, the rate of all complications of grade 3 or higher and of medical complications was similar between the two groups. The pattern of postoperative complications in the AGC-lap and EGC-lap groups was broadly similar (Table 4).

**Table 4:** Short-term outcomes (postoperative complications)

	EGC-lap (*n* = 41)		AGC-open (*n* = 37)		AGC-lap (*n* = 39)	
	All grades	≥ Grade 3	All grades	≥ Grade 3	All grades	≥ Grade 3
**Total complications**	5 (12.2)	2 (4.9)	16 (43.2)	7 (18.9)	7 (17.9)*	5 (12.8)
**Surgical complications**	3 (7.3)	2 (4.9)	14 (37.8)	4 (10.8)	4 (10.3)*	4 (10.3)
Bleeding	0 (0)	0 (0)	1 (2.7)	1 (2.7)	3 (7.7)	3 (7.7)
Ileus/gastroparesis	0 (0)	0 (0)	4 (10.8)	0 (0)	0 (0)*	0 (0)
Wound infection	0 (0)	0 (0)	2 (5.4)	0 (0)	0 (0)	0 (0)
Intra-abdominal abscess	1 (2.4)	1 (2.4)	5 (13.5)	3 (8.1)	0 (0)*	0 (0)
Anastomotic leakage	0 (0)	0 (0)	3 (8.1)	2 (5.4)	1 (2.6)	1 (2.6)
Anastomotic stenosis	1 (2.4)	1 (2.4)	0 (0)	0 (0)	0 (0)	0 (0)
Lymphorrhea	1 (2.4)	0 (0)	0 (0)	0 (0)	0 (0)	0 (0)
**Medical complications**	2 (4.9)	0 (0)	2 (5.4)	2 (5.4)	3 (7.7)	1 (2.6)
Pneumonia	2 (4.9)	0 (0)	2 (5.4)	2 (5.4)	2 (5.1)	1 (2.6)
Pulmonary embolism	0 (0)	0 (0)	0 (0)	0 (0)	0 (0)	0 (0)
Cardiac complications	0 (0)	0 (0)	0 (0)	0 (0)	0 (0)	0 (0)
Ischemic stroke	0 (0)	0 (0)	0 (0)	0 (0)	0 (0)	0 (0)
Deep vein thrombosis	0 (0)	0 (0)	0 (0)	0 (0)	0 (0)	0 (0)
Others	0 (0)	0 (0)	0 (0)	0 (0)	1 (2.6)	0 (0)

*EGC* early gastric cancer, *AGC* advanced gastric cancer, *Lap* laparoscopic surgery

*p < 0.05, AGC-lap versus AGC-open

**Fig. 3:**
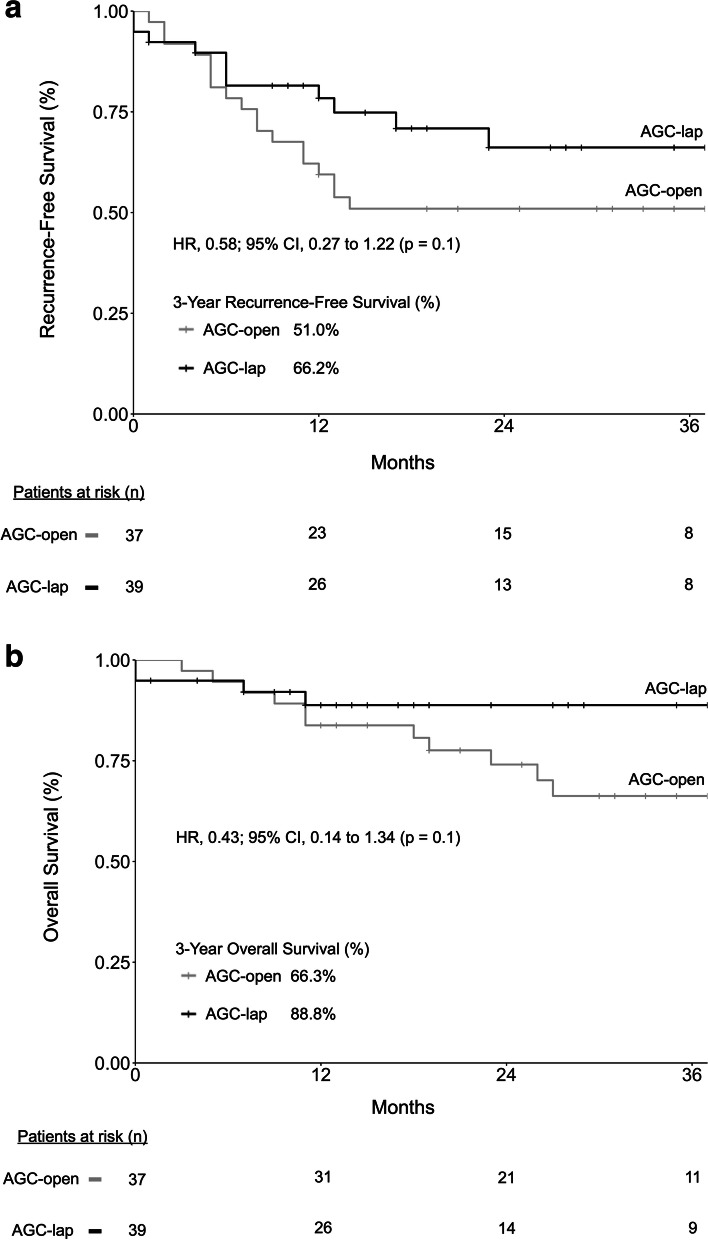
Kaplan–Meier analysis of 3-year recurrence-free survival and overall survival for AGC-lap versus AGC-open groups (**a**, **b**). Abbreviations: AGC, advanced gastric cancer; Lap, laparoscopic surgery

Three-year RFS was 66.2% in the AGC-lap group and 51.0% in the AGC-open group (Fig. 3a), and 3-year OS was 88.8% and 66.3%, respectively (Fig. 3b). The HRs for recurrence (or all-cause mortality) and all-cause mortality in the AGC-lap group compared to the AGC-open group were 0.58 (95% CI: 0.27–1.22; *p* = 0.1) and 0.43 (95% CI: 0.14–1.34; *p* = 0.1), respectively.

## Discussion

To our knowledge, this is one of few studies to comprehensively compare short-term results and oncological outcomes between LDG and ODG for elderly AGC patients. Furthermore, we included elderly EGC patients receiving LDG as a control group. We found that, although operative time was significantly longer in the AGC-lap group, the number of harvested lymph nodes was similar in AGC-lap and AGC-open surgeries. Notably, AGC-lap patients had a significantly shorter hospitalization, lower complication rates, and slightly better 3-year RFS/OS than AGC-open patients. As expected, the AGC-lap group differed significantly from the EGC-lap group in pathological features; however, the number of lymph nodes harvested, operative time, length of hospitalization, and complication rates were very similar.

Despite the fact that LDG is gradually being accepted and used more frequently for AGC, the role of LDG in elderly AGC patients has seldom been discussed and is poorly accounted for in large randomized controlled trials and meta-analyses [12–15]. In both the CLASS-01 and KLASS-02 trials, clinical T2–T4a GC patients with ECOG 0 or 1 and no bulky lymph nodes were included to compare the effects of LDG and ODG. The median age in the CLASS-01 trial was 57 years, and in the KLASS-02 trial, it was approximately 60 years [12–14]. In contrast, in this study, the median age was 77 years old, and approximately 30% of patients had ECOG > 1. Apart from age and performance, other surgical and pathological features in this study were comparable to those in the CLASS-01 and KLASS-02 trials. In AGC-lap patients, the mean number of lymph nodes harvested was 36.1 and 46.8 in the CLASS-01 and KLASS-02 trials, respectively, compared to a median of 37 in this study. Mean tumor size was 4 cm and 4.6 cm in the CLASS-01 and KLASS-02 trials, respectively, compared to a median of 4.5 cm in this study. The percentage of clinical node-positive disease was around 50%. The mean operative time and blood loss in AGC-lap patients were 227 min and 152.4 mL, respectively, in the KLASS-02 trial, which are similar to the median values in this study (215 min and 100 mL).

AGC and EGC were often grouped together to analyze the effects of LDG and ODG in elderly GC patients; however, the differences in disease severity and surgical technique requirement between EGC and AGC may introduce some biases to study results. Among the limited number of retrospective studies related to this study, elderly EGC patients were often included and ranged between 14.8 and 41.4% of the study population [17, 36]. Additionally, elderly patients receiving less than a D2 lymphadenectomy were also included in some studies. For example, in a propensity-score matching study of a cohort with a median age of 71 years, 64.4% and 44.2% of the LDG and ODG groups, respectively, were EGC cases, and only 80% of patients received D2 gastrectomy [18]. The separation of AGC and EGC patients, all of whom received D2 gastrectomy by LDG or ODG, in this study may therefore make it more objective than other retrospective studies.

Similar to previous studies, LDG was associated with significantly longer operative times but shorter postoperative hospitalization than ODG. The number of harvested lymph nodes was similar, which suggests similar surgical qualities [14, 17, 19, 36]. The longer operative time in LDG raised concerns regarding the negative effects of the prolonged pneumoperitoneum required for precise dissection [37, 38]. We therefore compared operative time, length of hospitalization, and number of harvested lymph nodes between AGC- and EGC-lap groups, since LDG is a well-established and standard approach for EGC, even in elderly patients [28, 29]. We expected AGC-lap patients to have significantly longer operative times, more blood loss, and longer hospitalization due to the complex nature of the disease and pneumoperitoneum-related complications. However, the median duration of hospitalization and blood loss were 11 days and 100 mL in both groups, and the median operative time was 203 and 215 min in the EGC- and AGC-lap groups, respectively (Table 3). This indicates highly similar surgical characteristics between these two groups, a relationship rarely documented. These similarities may promote the gradual development and increased use of LDG for AGC and elderly patients, as surgical techniques improve and familiarity with anatomy and postoperative care develops, as was the case in our surgical team (Fig. 2) [4].

Postoperative complications, especially surgical ones, regardless of grading, occurred significantly more frequently in the AGC-open group than in the AGC-lap and EGC-lap groups (Table 4). Complications related to medical conditions were similar between AGC-lap and AGC-open groups and between the AGC- and EGC-lap groups. The most common surgical complications of all grades in the AGC-open group were intra-abdominal abscess (13.5%), followed by ileus/gastroparesis (10.8%). The total complication rates in the KLASS-02 trial in AGC-open and AGC-lap patients were 32.9% and 20.1%, respectively (*p* < 0.05), and the total surgical complication rates were 22.2% and 14.2% (*p* < 0.05) [14]. Kim et al. reported a 22.0% total complication rate in laparoscopic gastrectomy for patients aged ≥ 80 years, compared to 30.1% in open gastrectomy (*p* = 0.249) [17]. Open gastrectomy was found to be a significant risk factor (odds ratio = 2.49; *p* = 0.049) for postoperative complications after further multivariable analyses adjusted for age, CCI, and tumor status (EGC or AGC) [17]. Chen et al. also reported a significantly higher surgical complication rate in open gastrectomy (20.9%) than in laparoscopic gastrectomy (9.3%) for elderly patients (*p* = 0.009), comprising mostly ileus/gastroparesis (6.2%), intra-abdominal abscess (4.7%), and anastomotic leak (3.1%) [18]. This study demonstrates the complication profile similar to previous studies and indicates that LDG could be safely applied to elderly AGC patients [19].

Survival data on elderly AGC patients after surgery are limited and rarely reported properly. Multiple factors, including age, preoperative performance status, comorbidities, cancer staging, surgical method, complications, and adjuvant chemotherapy influence long-term outcomes. Three-year RFS and OS were both better in the AGC-lap than in the AGC-open group, though not significantly (RFS: 66.2% versus 51%; OS: 88.8% versus 66.3%; both *p* = 0.1; Fig. 3). In the CLASS-01 trial, 3-year RFS was 76.5% and 77.8%, and 3-year OS was 83.1% and 85.2%, in LDG and ODG patients, respectively. Similarly, in the KLASS-02 trial, RFS was 80.3% and 81.3%, and OS was 90.6% and 90.3%, in the LDG and ODG groups, respectively. The survival rates in these two highly relevant prospective trials were thus significantly better than those in our study; however, the patients in these studies were around 20 years younger on average, and 30–35% of those cases were EGC. The percentages of patients receiving adjuvant chemotherapy after radical gastrectomy were highly various among studies, especially in the elderly population. The adjuvant chemotherapy rate was 41% in this study (AGC-lap versus AGC-open: 46% versus 35%; *p* = 0.4), 39.4% in the CLASS-01 trial, and 61.3% in the KLASS-02 trial [12–14]. In a retrospective study focusing on elderly GC patients, comparable 3-year RFS (laparoscopic versus open gastrectomy: 67% versus 69.5%) and OS (70.1% versus 70.8%) rates were reported [18]. However, approximately 40% of these cases were EGC and the adjuvant chemotherapy rate was not reported [18]. The efficacy and cost-effectiveness of adjuvant chemotherapy in elderly patients should always be evaluated carefully and discussed thoroughly prior to initiation; thus, the decisions are often difficult considering advanced age and multiple comorbidities in this population [24, 25]. Further study may be required to elaborate this issue.

The main strength of our study was the inclusion of a well-defined elderly AGC patient cohort, all of whom received LDG or ODG with standard D2 lymphadenectomy by the same surgical team, from a prospectively collected database, reducing bias and ensuring consistency in surgical technique, quality of postoperative care, and follow-up policy. The primary aim was to better understand the short-term and oncological outcomes of LDG compared to ODG in elderly AGC patients. We also included elderly EGC patients receiving LDG, for comparison with the AGC patients, providing a more complete picture, especially in terms of short-term outcomes. Nevertheless, our study had limitations. Firstly, selection bias may have existed between the AGC-lap and AGC-open groups, although there were no significant differences in baseline and pathological features. The percentage of clinical N+ stage patients, tumor size, and Borrmann type III or IV lesions was slightly greater in the AGC-open group. Secondly, the temporal distribution of AGC-lap and AGC-open cases was uneven. In the first few years of the study, open surgery was more common than laparoscopic surgery; thus, follow-up was significantly shorter for the AGC-lap group. Finally, the sample size was relatively small. Owing to the difficulty and uncertainty in conducting clinical trials on elderly AGC patients [16, 19], increasing case numbers and conducting further longitudinal follow-up may be crucial to obtain more robust conclusions.

## Conclusions

Our study clearly reveals short-term and oncological outcomes for elderly AGC-lap patients via comparison with AGC-open and EGC-lap patients. Although involving significantly longer operative times, AGC-lap patients benefited from significantly shorter postoperative hospitalization and fewer complications than AGC-open patients. The use of LDG in elderly AGC patients scarcely increased operative time, hospitalization duration, or complication rates relative to elderly EGC patients. Three-year RFS and OS were similar in the AGC-lap and AGC-open groups.

## Data Availability

The datasets generated and/or analyzed during the current study are not publicly available but are available from the corresponding author on reasonable request.

## Abbreviations

GC: Gastric cancer
EGC: Early gastric cancer
AGC: Advanced gastric cancer
LDG: Laparoscopic distal gastrectomy
ODG: Open distal gastrectomy
RCT: Randomized controlled trial
lap: Laparoscopic
EGD: Esophagogastroduodenoscopy
ECOG: Eastern Cooperative Oncology Group
ASA: American Society of Anesthesiologists
CCI: Charlson comorbidity index
GNRI: Geriatric nutritional risk index
PNI: Prognostic nutrition index
RFS: Recurrence-free survival
OS: Overall survival
IQR: Interquartile range
HR: Hazard ratio
CI: Confidence interval
